# Determinants of Medication Storage and Disposal Practices in Brazil: A Cross-Sectional Study with Implications for Environmental Exposure

**DOI:** 10.3390/epidemiologia7040090

**Published:** 2026-06-29

**Authors:** Jorge Fernando Carrozza, Clovis Bergamin Griso, Rafaela Felix Vieira Bastos, Gabriel Leandro Jesus Santos, Marcela Cristina Garnica-Siqueira, Gabriela Moraes Oliveira, Thiago José Dionísio, Carlos Ferreira Santos, Adriana Maria Calvo

**Affiliations:** 1 Department of Biological Sciences, Bauru School of Dentistry, University of São Paulo, Bauru 17012-901, SP, Brazil; jorgecarrozza@usp.br (J.F.C.); clovis.bergamin@usp.br (C.B.G.); gabriel.jesuss@usp.br (G.L.J.S.); gab.moraes@usp.br (G.M.O.); thiagoj@usp.br (T.J.D.); cfsantos@fob.usp.br (C.F.S.); 2 Bauru School of Medicine, University of São Paulo, Bauru 17012-901, SP, Brazil; rafaelafelix@usp.br (R.F.V.B.); mcgsiqueira@usp.br (M.C.G.-S.); 3 Hospital for Rehabilitation of Craniofacial Anomalies, University of São Paulo (HRAC/USP), Bauru 17012-901, SP, Brazil

**Keywords:** healthcare waste, medication disposal, medication storage, pharmaceuticals, environmental pollution, environmental sustainability, public awareness

## Abstract

Background/Objectives: Inappropriate medication storage and disposal are recognized public health concerns and represent a relevant source of environmental exposure to pharmaceutical compounds, with potential implications for ecosystem and human health. Identifying population-level determinants of these behaviors is essential to support targeted interventions and risk mitigation strategies. Methods: A cross-sectional population-based study was conducted using a structured survey administered to Brazilian adults. The questionnaire assessed medication storage practices, disposal behaviors, and prior exposure to guidance on appropriate medication handling. Descriptive statistics were used to characterize the sample, and multivariate logistic regression analysis was performed to identify independent predictors of appropriate disposal practices. Results: A total of 475 participants were included in the analysis. Although awareness of the environmental impact of improper medication disposal was high, inappropriate practices remained prevalent. Multivariate logistic regression identified educational level as the primary independent determinant of appropriate disposal practices (*p* < 0.001), while no significant associations were observed for age or gender (*p* > 0.05). Lack of prior guidance was frequent and may represent a relevant population-level exposure influencing unsafe behaviors. Conclusions: The findings highlight a gap between awareness and practice and identify key sociodemographic determinants influencing medication disposal behaviors. From an environmental epidemiology perspective, improper disposal may contribute to the dissemination of pharmaceutical residues in water systems, representing an indirect but widespread exposure pathway. Targeted public health strategies focusing on high-risk groups are needed to promote safe medication handling and reduce environmental and health risks.

## 1. Introduction

The use of medications, whether prescribed or over-the-counter, is growing every day worldwide; as a result, more medications are expected to accumulate or be left unused at home [1,2,3,4]. This also applies to their packaging, whether plastic, leaflets, or cardboard. Unused medications end up being stored at home, discarded in household waste, or flushed down the drain or toilet [5,6,7]. Sharps materials, including syringes and needles [8], and more recently, medications administered via injection for weight loss and diabetes management, have contributed to a worsening trend.

In this critical scenario, a significant public health concern emerges as the environment is receiving a large amount of contaminating substances, mainly medications and their residues, which are called “emerging contaminants” [9,10].

Considering the environmental and public health implications of pharmaceutical waste, this study is particularly aligned with Sustainable Development Goal (SDG) 3 (Good Health and Well-Being), SDG 6 (Clean Water and Sanitation), and SDG 12 (Responsible Consumption and Production) established by the United Nations [11].

**Figure 1:**
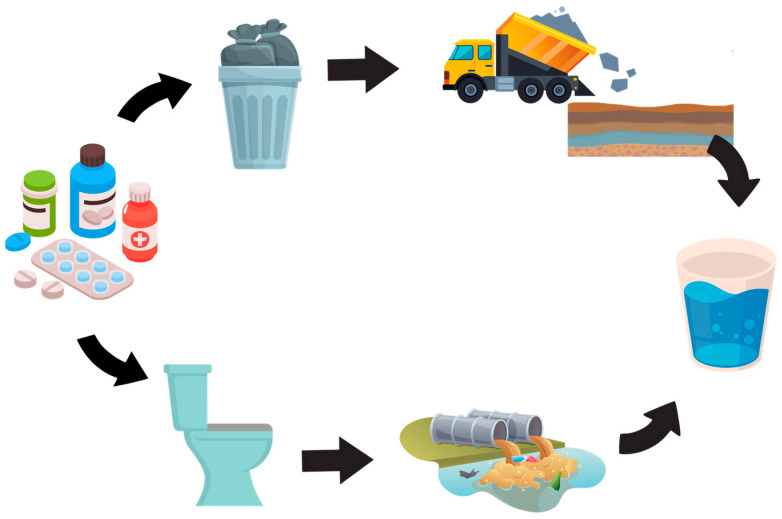
The life cycle of improperly discarded medications.

Storing medications at home for temporary conditions like headache, fever, or heartburn can be called a “home pharmacy” [10,12,13]. Discontinuation of treatment after the resolution of symptoms is also a source of increased medication in this “home pharmacy.” Epidemiological studies conducted in southern Brazil showed that >90% of respondents stored medications at home [12,14]. Many of these medications in the “home pharmacy” tend to be out of date and are disposed of in different ways, mainly through unsafe disposal methods such as sinks, toilets, or household waste and garbage, which can harm the environment and human health [15] (Figure 1).

Regarding household storage locations, the stability and safety of medications stored in “home pharmacies” should also be considered. Previous studies have identified kitchens, bedrooms, medicine cabinets, and bathrooms as the most commonly reported storage locations. However, many of these environments may expose medications to humidity, light, temperature fluctuations, or inappropriate access by children, potentially compromising medication stability and safety [16,17].

Expired and unused medications constitute pharmaceutical waste [4]. Unused medications can be used by other patients; however, for safety reasons (there is a global problem with counterfeit medications), such medications can only be dispensed by a licensed pharmacy. The main reasons why a medication becomes unusable by a patient or eventually remains unused are (1) a change in dosage or medication replacement; (2) the patient’s death; (3) failure to complete therapy or inappropriate medication use by the patient (especially antibiotics); and (4) discontinuation of therapy due to side effects [18].

Inappropriate use and premature discontinuation of antibiotic therapy represent important public health concerns, as they may contribute to antimicrobial resistance and reduce the effectiveness of available treatments [19]. The accumulation of leftover antibiotics in households may further facilitate self-medication and inappropriate reuse practices.

Medication disposal is a significant public health concern, classified by the Brazilian Health Regulatory Agency (Anvisa) as Class B waste. This waste can be considered toxic depending on its composition. Healthcare waste consists of medications, contaminated syringes, antibiotics, and other potentially infectious materials, which can harm the environment and the individuals who come into contact with them [20]. This also poses a significant challenge for patients who use injectable medications, such as diabetics, or those with other comorbidities who require daily injections.

Enhancing global awareness among the general population and healthcare professionals regarding the importance of the proper disposal of unused or unwanted medications is urgently needed. Research is needed to assess pharmacists’ attitudes and methods for disposal in pharmacies and other collection points [21]. This is in addition to raising public awareness about proper disposal. Approximately half of the respondents in a survey by Tegegne et al. [22] stated that they do not check the expiration date of their regularly consumed medications.

A Brazilian study published in 2024 [23] found that 54.4% of respondents did not know whether their cities had specific collection points for medications. Although the National Solid Waste Policy, published in 2010 in Brazil, stipulated that waste would be returned to its original producers [24], a large percentage of respondents (71.9%) never received any information about proper medicine disposal. These figures are primarily due to the lack of a Brazilian reverse logistics system combined with low investment in environmental education to inform the population [23], which highlights an important public health concern.

Despite the implementation of the Brazilian National Solid Waste Policy and recent reverse logistics initiatives for pharmaceutical waste, adherence to appropriate medication disposal practices remains limited in Brazil, and epidemiological data on household storage and disposal behaviors are still scarce and regionally concentrated [23,24]. In Brazil, household pharmaceutical waste collection programs are primarily based on collection points located in pharmacies, drugstores, and some healthcare facilities, although accessibility and public awareness remain heterogeneous across different regions of the country. Furthermore, few studies have simultaneously evaluated medication storage, disposal practices, environmental awareness, and educational interventions within the Brazilian context. Therefore, understanding the determinants associated with these behaviors may support the development of targeted public health and environmental strategies.

## 2. Materials and Methods

The survey in this cross-sectional study involved a questionnaire on how the population stores and disposes of unused and/or expired medications, sharps resulting from the use of injectable medications, and plastic and cardboard packaging. The survey also addressed questions about the level of information the population had on these topics and their opinions on how this information could be better disseminated.

In parallel, educational activities were carried out in public and private schools in the city of Bauru, in the interior of the state of São Paulo, Brazil, as well as in open-air markets and places with high traffic across the city, with the distribution of educational pamphlets and lectures.

This study was approved by the Human Research Ethics Committee of the Bauru School of Dentistry (CEP/FOB/USP—CAAE: 88129225.1.0000.5417) and only began after its approval.

### 2.1. Target Population

The electronic questionnaire (via Google Forms) was sent to individuals over 18 years of age and residing in Brazil who wanted to participate in the study. The questionnaire remained accessible to any adult residing in Brazil, regardless of nationality.

Before beginning the questionnaire, participants were required to accept the Informed Consent Form to continue the response process. They were free to withdraw or withdraw their responses at any time, either before the final submission or after submitting the form.

### 2.2. Questionnaire

The invitation to participate in this cross-sectional survey was disseminated through open social media sharing by researchers and collaborators using platforms such as Instagram, Facebook, and WhatsApp. Participation was voluntary and unrestricted to adults over 18 years of age residing in Brazil, regardless of nationality. In parallel, posters and informational pamphlets containing a QR code linked to the questionnaire were distributed in high-traffic public locations in Bauru, Brazil. Although these printed materials were distributed locally, the online questionnaire remained openly accessible nationwide throughout the data collection period.

Participants provided information regarding sociodemographic characteristics, medication storage and disposal practices, handling of injectable materials and packaging, environmental awareness, and previous exposure to guidance regarding pharmaceutical waste management. The complete questionnaire is available as Supplementary Material (Supplementary File S1).

Participants’ personal data were kept strictly confidential and used only for the purposes of this study. Identification document numbers were requested exclusively to minimize duplicate submissions and were not used for participant identification, eligibility assessment, or statistical analysis.

### 2.3. Variable Classification and Outcome Definitions

Initially, the raw results were described, with percentage distributions across all participant responses, since in several questionnaire questions, it was possible to select more than one option, especially regarding the storage and disposal of medications.

Subsequently, dichotomizations were performed to allow for statistical correlation analysis. Adequate storage was defined as keeping medications in indoor environments protected from excessive humidity, light exposure, and temperature fluctuations, such as bedroom cabinets or storage boxes. Inadequate storage included locations subject to environmental instability, such as kitchens, bathrooms, and mobile storage (e.g., bags or backpacks). For questions allowing multiple responses, a conservative approach was applied, whereby the presence of any unsafe storage condition resulted in classification as inadequate.

Adequate disposal was defined exclusively as returning medications to officially designated collection points, including pharmacies, healthcare facilities, or authorized public collection programs. All other disposal methods, such as disposal in household waste, sinks, or toilets, were classified as inadequate due to their potential environmental impact.

For analytical purposes, medication packaging was categorized as primary packaging (materials with potential exposure to pharmaceutical residues, such as blisters, bottles, tubes, and vials) and secondary packaging (external cardboard boxes and package inserts). Primary packaging was considered potentially contaminated due to possible exposure to pharmaceutical residues.

For regression analysis, categorical variables were numerically coded. Gender was coded as 0 (female) and 1 (male). Age group was converted into ordinal categories (1–4), corresponding to increasing age ranges. Educational level was dichotomized into lower education (0) and higher education (1), based on the presence of tertiary or postgraduate education.

### 2.4. Statistical Analysis

Data were analyzed using GraphPad Prism software (version 8.4.3). Associations between variables were assessed using chi-square or Fisher’s exact tests, as appropriate. Multivariate logistic regression models were applied to evaluate predictors of appropriate medication disposal. The model included gender, age group, educational level, and prior guidance as independent variables. Prior guidance referred to any previous information or orientation regarding appropriate medication storage or disposal practices, regardless of source (e.g., healthcare professionals, educational campaigns, pharmacies, or media sources). Adjusted odds ratios (ORs) with 95% confidence intervals (CIs) were calculated. A significance level of 5% was adopted for all analyses.

## 3. Results

### Sociodemographic Data

A total of 475 surveys were completed, with most responses submitted by female participants (67.6%). The predominant age group was 18–28 years (49.7%). Educational attainment was heterogeneous, with complete higher education being the most frequent category (31.2%), followed by incomplete higher education (29.50%) and postgraduate, master’s, and PhD (18.5%). Lower educational levels were underrepresented, including incomplete elementary education (0.4%) and complete elementary education (1.1%). Regarding geographic distribution within Brazil, the majority of participants were from the Southeast region (89.2%), followed by the Central-West (5.3%) and North and Northeast (1.1% each). The demographic characteristics of the participants are summarized in the table below (Table 1).

**Figure 2:**
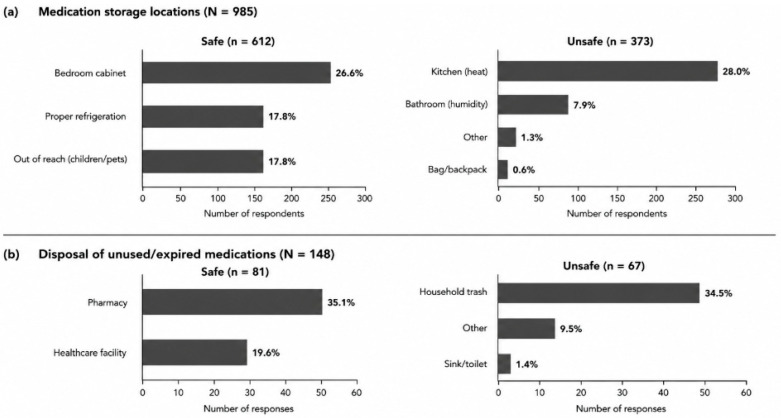
Combined overview of household medication management practices: (**a**) Reported medication storage locations according to safety criteria. (**b**) Reported disposal locations for unused or expired medications, categorized as safe or unsafe. Values beside bars are shown as absolute counts and percentages. For storage, percentages were calculated using the total number of storage-location mentions (*n* = 985), since respondents could select more than one option. For disposal practices, percentages were calculated using the total number of categorized disposal responses (*n* = 148).

Medication storage practices varied considerably among participants, and the questionnaire allowed for multiple responses per individual. Overall, safe storage locations accounted for the majority of reported mentions (62.1%), whereas unsafe locations represented 37.9%. The most commonly reported safe practice was storage in bedroom cabinets or storage boxes (26.6%), followed by keeping medications out of reach of children and animals (17.8%) and appropriate refrigeration (17.8%), suggesting a moderate level of awareness regarding recommended storage conditions. However, a substantial proportion of participants reported storing medications in potentially unsuitable environments. Kitchen storage was the most frequently reported unsafe practice (28.0%), followed by bathroom storage (7.9%), both of which are characterized by temperature fluctuations and humidity that may compromise drug stability. Less frequent unsafe locations included bags or backpacks (0.6%) and unspecified locations (1.3%) (Figure 2a).

Practices for disposing of unused medications presented a concerning pattern, with unsafe disposal behaviors representing a substantial proportion of responses. Among safe disposal methods, medication collection programs at pharmacies were the most frequently reported (35.1%), followed by disposal at healthcare facilities (19.6%). However, unsafe practices were also highly prevalent, particularly disposal in household waste (34.5%), which was almost as frequent as disposal at pharmacies. Other inadequate methods included unspecified disposal routes (9.5%) and disposal in sinks, toilets, or drains (1.4%), which can contribute to environmental contamination. These findings indicate that, despite the availability of adequate disposal routes, a significant portion of individuals continue to rely on convenient but unsafe methods, reinforcing the need for targeted educational and structural interventions (Figure 2b).

After applying context-adjusted classification criteria and for analytical purposes, 46.5% of participants were classified as having adequate medication storage practices, while 53.5% reported at least one inadequate condition. No statistically significant associations were observed between storage practices and sociodemographic variables, including gender, age group, and educational level (*p* > 0.05 for all comparisons). These findings suggest that storage practices were relatively homogeneous across the study population.

**Figure 3:**
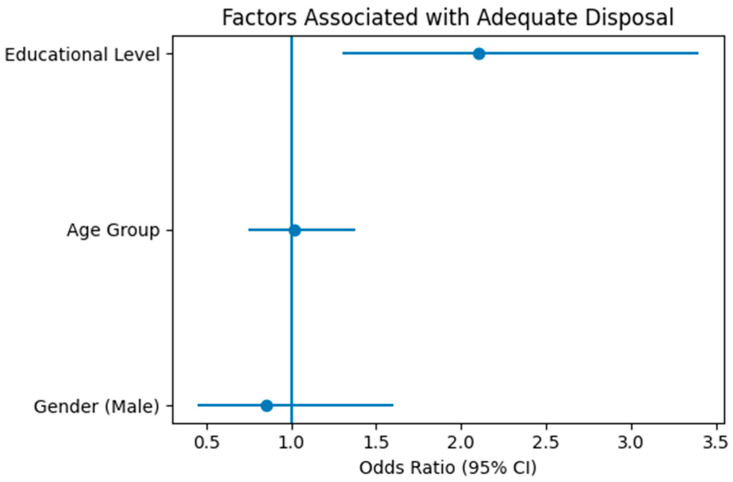
Logistic regression analysis (forest plot). Odds ratios and 95% confidence intervals for factors associated with adequate disposal practices.

Since the same participant could select more than one answer, at the end of the dichotomization of the results, proper disposal of medications was reported by 22.4% of participants, while 77.6% reported improper disposal practices. A statistically significant association was observed between educational level and disposal practices (*p* < 0.001). Participants with higher educational levels had higher odds of appropriate medication disposal compared to those with lower educational levels (Figure 3). No significant associations were identified between disposal practices and gender or age group (*p* > 0.05).

Most participants reported periodically checking the expiration dates of their medications (69.6%), but the vast majority (76.2%) reported keeping medications from treatments that have already been completed. Of the participants, 54.5% use continuous medication, with the majority indicating the use of only one medication (22.1%) or two to five medications (30.4%). The use of injectable medications was low (4.7%); however, among these individuals, most reported proper disposal practices (81.8%). Notably, nearly all participants recognized that improper medication disposal may harm the environment (97.5%) and believed that the general population lacks sufficient knowledge on this topic (98.8%), despite only 35.9% reporting having previously received guidance (Table 2), highlighting a substantial gap between awareness and formal education.

**Figure 4:**
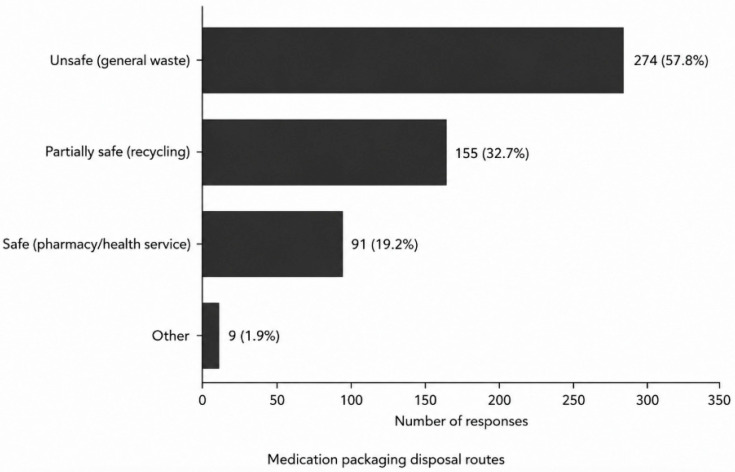
Reported disposal routes for medication packaging. Responses were classified according to disposal practice as safe (return to pharmacy or healthcare service), partially safe (recycling), unsafe (general household waste), or other. Data are presented as absolute counts and percentages. Because participants could report more than one disposal route, categories were not mutually exclusive.

Regarding packaging, both secondary packaging, such as boxes and leaflets that could easily be recycled, and primary packaging, such as blisters and jars (glass or plastic), were also investigated regarding their final destination. Unsafe disposal of general waste was the most frequently reported route for medication packaging, mentioned by 274 participants (57.8%). Recycling was the second most common route, reported by 155 participants (32.7%), whereas return to pharmacies or health services was reported by 91 participants (19.2%). Responses classified as other were uncommon (1.9%). Percentages were calculated based on the total number of respondents (*n* = 474). As participants could report more than one disposal route, categories were not mutually exclusive (Figure 4).

A high level of awareness regarding the environmental risks associated with improper medication disposal was observed among participants. However, disposal practices remained heterogeneous, with a substantial proportion of responses indicating disposal in household waste.

In parallel with these findings, the project included an educational outreach component aimed at promoting the safe use, storage, and disposal of medications. Approximately 500 elementary and high school students (public and private) participated in in-person lectures. An Instagram page (@descarteconscientefobusp) was created to share educational posts with the general public. In addition, around 8000 educational leaflets were distributed during community initiatives, including street markets, science and technology fairs, and cultural events held in Bauru, broadening the dissemination of information to a wider population.

## 4. Discussion

### 4.1. Public Health and Environmental Implications

This study provides epidemiological evidence on medication storage and disposal behaviors in a Brazilian population, identifying key sociodemographic determinants associated with unsafe practices. Despite high levels of awareness regarding environmental risks, a substantial proportion of participants reported inappropriate disposal behaviors, indicating a persistent gap between knowledge and practice. From an environmental epidemiology perspective, improper medication disposal may contribute to the continuous release of pharmaceutical residues into water systems, representing an indirect but widespread exposure pathway. Although individual-level contributions may appear limited, the cumulative population effect may be substantial, particularly in settings with limited disposal infrastructure.

The safe management of pharmaceutical waste is increasingly recognized as a critical interface between public health and environmental sustainability. In this context, the findings of the present study align with the principles of the United Nations Sustainable Development Goals (SDGs), particularly SDG 3 (Good Health and Well-Being), SDG 12 (Responsible Consumption and Production), and, indirectly, SDG 6 (Clean Water and Sanitation) [11]. By addressing the most common household practices related to the storage and disposal of medications and their packaging, this study contributes to the understanding of how individual behaviors influence environmental contamination, the rational use of medications, and sustainable waste management systems.

### 4.2. Household Storage and Disposal Behaviors

The results indicate that home medication management is characterized by partial adherence to recommended practices, with a clear imbalance between relatively safer storage behaviors, such as keeping medications out of reach of children and animals, and less appropriate disposal practices, such as disposal in regular trash. Although safe storage locations predominated, a considerable proportion of participants still reported storing medications in kitchens and bathrooms, environments known to compromise medication stability due to heat, humidity, and light exposure [17,25]. Similar patterns have been reported in recent studies, where inappropriate storage conditions remain common despite general awareness of recommended practices worldwide [1,2,4,5,13,15]. These findings reinforce that storage behavior is strongly influenced by convenience and routine rather than exclusively by knowledge.

From a public health perspective, improper storage also represents a relevant safety concern [17]. Medications kept within easy reach or in mobile locations such as bags and backpacks may increase the risk of unintentional exposure, particularly among children. According to the U.S. Centers for Disease Control and Prevention (CDC), epidemiological data indicate that, regarding accidental poisoning, approximately 100 young children are taken to the emergency room every day after ingesting medications that were within their reach. These include commonly used medications, vitamins, and other supplements, including those most attractive to them, in gummy/candy form. The worldwide recommendation is that all of these should be kept out of the reach and sight of young children. This also includes the elderly, who are another highly vulnerable group. Recent evidence continues to highlight the association between unsafe household storage and accidental poisoning events, emphasizing the importance of preventive educational strategies [13,17].

In contrast to storage practices, disposal behaviors revealed more significant limitations. While pharmacies and healthcare facilities were identified as appropriate disposal routes, disposal in household waste was reported with comparable frequency. This finding in our study is consistent with recent cross-sectional studies demonstrating that improper disposal—particularly through household waste—remains one of the most prevalent practices globally, even in populations with moderate awareness of environmental risks [1,5,15,22]. These patterns suggest that accessibility and convenience are key determinants of behavior, often overriding environmental considerations, which represents an increasing public health and environmental concern.

### 4.3. Sociodemographic Determinants and Behavioral Factors

Multivariate logistic regression analysis confirmed that educational level was the only significant predictor of appropriate medication disposal. Individuals with higher educational levels had higher odds of appropriate disposal behavior, while no significant associations were observed for gender or age group. These findings suggest that disposal practices may be more strongly influenced by access to information and cognitive factors than by demographic characteristics. Similar patterns have been reported in previous studies, in which appropriate disposal behaviors were associated with educational attainment, whereas other sociodemographic variables showed limited influence [1,13]. These findings reinforce that educational attainment plays a central role in shaping appropriate disposal behavior, whereas demographic characteristics alone appear insufficient to explain variability in these practices.

Another relevant finding was the high prevalence of unused medications stored at home, whether to share with family and friends or to keep for a future illness, even among individuals who reported routinely checking expiration dates. This apparent contradiction has also been observed in recent literature, where the accumulation of unused medications is attributed to changes in treatment, resolution of symptoms, and preventive storage practices [4,13]. The persistence of these “home pharmacies” represents a contributing factor to medication waste, facilitating self-medication, the development of bacterial resistance, inappropriate reuse, and late disposal.

Although the proportion of participants using injectable medications was low, most reported proper disposal of sharps. While this may reflect a higher perception of risk associated with these materials, the limited sample size requires cautious interpretation, as these participants often receive injectable medication at health centers where more structured guidance on the dangers of improper disposal is provided. Nevertheless, with the increasing use of injectable therapies for chronic diseases and obesity, improper disposal of sharps remains a relevant concern due to the potential for injury and contamination [8]. Catic and colleagues studied the disposal patterns of used pens and needles from diabetic patients in Bosnia and Herzegovina, and the numbers in their country indicated substantial deficiencies in disposal knowledge, demonstrating a lack of knowledge about the most appropriate place for their disposal [26].

One of the most significant findings of this study is the clear discrepancy between environmental awareness and actual behavior. Although almost all participants recognized the environmental risks associated with the improper disposal of medications and admitted to insufficient public knowledge on the subject, only a minority reported having received formal guidance. This gap is widely reported in recent studies, which consistently demonstrate that awareness alone is insufficient to promote behavioral change, especially in the absence of structured education and accessible and visible disposal systems [1,22,26]. These findings highlight the need for interventions that go beyond disseminating information and actively facilitate behavioral change.

### 4.4. Pharmaceutical Packaging and Educational Outreach

The analysis of pharmaceutical packaging disposal further reinforces this issue. Secondary packaging, such as cardboard boxes and paper leaflets, may be suitable for conventional recycling, whereas primary packaging, including blisters and bottles with direct contact with medications, may represent a potential source of pharmaceutical contamination. Improper disposal of household waste was the most frequently reported practice, while recycling and return to healthcare services were less common. These findings suggest that medication packaging is often perceived as ordinary household waste despite its potential environmental implications. Recent environmental health studies highlight pharmaceutical packaging as an important, yet frequently neglected, component of healthcare waste streams [10], representing a potential environmental hazard [8].

It is important to highlight that the integration of an educational outreach component strengthens the translational relevance of this study. Conducting lectures in schools, disseminating information through social media, and distributing educational materials on a large scale directly addressed the information gaps identified in the research. Educational interventions targeting both young people and the community at large have been increasingly recognized as effective strategies for improving medication-related behaviors and promoting sustainable practices [22], reinforcing the importance of early educational interventions for future generations.

Taken together, these findings suggest that improving medication-related practices requires a multidisciplinary approach that goes beyond individual awareness. Educational initiatives should be combined with greater visibility of collection programs and disposal locations, clearer communication strategies, and stronger integration between public health systems, pharmacies, and environmental policies. Interventions based on community education, school-based activities, pharmacist counseling, and public awareness campaigns may be particularly effective because they increase both knowledge and accessibility to safe disposal practices. In the Brazilian context, strengthening reverse logistics systems and expanding geographically accessible collection points remain essential to ensure that safe disposal practices are both accessible and routinely adopted by the population.

### 4.5. Study Limitations and Strengths

This study has some limitations. The cross-sectional design precludes causal inference. In addition, the convenience sampling strategy and online dissemination may have introduced selection bias, potentially favoring participation of younger, more educated individuals and participants connected to academic or health-related social media networks. The demographic profile of the sample differed from the general Brazilian population, with a predominance of participants from the Southeast region and individuals with higher educational attainment, likely reflecting the online convenience sampling approach and dissemination methods. Therefore, the findings should be interpreted as indicative of behavioral trends rather than nationally representative prevalence estimates. Furthermore, self-reported behaviors may be subject to recall bias and social desirability bias, and residual confounding related to unmeasured socioeconomic or behavioral variables cannot be excluded. Despite these limitations, the study provides relevant insights into household medication storage and disposal behaviors and is strengthened by the integration of quantitative findings with real-world educational interventions.

## 5. Conclusions

This study demonstrates that, although environmental awareness regarding pharmaceutical waste is high, it does not consistently translate into safe storage and disposal practices. Unsafe disposal, particularly in household waste, remains frequent, while knowledge about appropriate disposal routes is limited, and formal guidance is insufficient. These findings highlight a critical gap between awareness and behavior, indicating that improving medication-related practices requires not only education but also accessible and visible disposal systems. Integrating public health strategies with environmental policies and community-based educational initiatives may be essential to promote safer and more sustainable pharmaceutical waste management.

## Figures and Tables

**Table 1:** Sociodemographic characteristics of the participants.

		*n*	Percentage (%)
Gender	Male	151	31.8
	Female	321	67.6
	Prefer not to answer	3	0.6
Age	18 to 28 years old	236	49.7
	29 to 48 years old	148	31.2
	49 to 68 years old	79	16.6
	69 years old and over	12	2.5
Education	Incomplete Elementary School	2	0.4
	Complete Elementary School	5	1.1
	Incomplete High School	6	1.3
	Complete High School	86	18.1
	Incomplete Higher Education	140	29.5
	Complete Higher Education	148	31.2
	Postgraduate, Master’s and PhD	88	18.5
Brazil Region	South Region	16	3.4
	Southeast Region	423	89.2
	Central-West Region	25	5.3
	North Region	5	1.1
	Northeast Region	5	1.1

Values are presented as *n* and valid %. Missing responses were excluded from each variable denominator (gender *n* = 475, age *n* = 475, education *n* = 475, region *n* = 474).

**Table 2:** Medication use, disposal practices, environmental awareness, and guidance-related characteristics.

		*n*	Percentage (%)
Checks medication expiry periodically (*n* = 474)	Yes	330	69.6
	No	144	30.4
Keeps leftover medication (*n* = 475)	Yes	362	76.2
	No	113	23.8
Uses continuous medication (*n* = 470)	Yes	258	54.5
	No	215	45.5
	1	104	22.1
	2 to 5	143	30.4
	6 or more	15	3.2
Uses injectable medication (*n* = 271)	Yes	22	4.7
	No	449	95.3
Proper disposal of injectable medications (*n* = 22)	Yes	18	81.8
	No	4	18.2
Believes improper disposal harms the environment (*n* = 275)	Yes	463	97.5
	No	12	2.5
Previously received guidance (*n* = 273)	Yes	170	35.9
	No	303	64.1
Believes the population lacks sufficient knowledge (*n* = 275)	Yes	474	98.8
	No	1	0.2

Values are presented as *n* and valid %. Missing responses were excluded from each variable denominator.

## Data Availability

An Instagram page was created to raise public awareness about the responsible disposal of medications: @descarteconscientefobusp.

## Supplementary Materials

The following supporting information can be downloaded at: https://www.mdpi.com/article/10.3390/epidemiologia7040090/s1. The complete questionnaire is available as Supplementary File S1.

## Abbreviations

The following abbreviations are used in this manuscript:

SDGs	Sustainable Development Goals
Anvisa	Brazilian Health Regulatory Agency
CEP/FOB/USP	Human Research Ethics Committee of the Bauru School of Dentistry
FOB/USP	Bauru School of Dentistry/University of São Paulo
HRAC/USP	Hospital for Rehabilitation of Craniofacial Anomalies/University of São Paulo
CDC	Centers for Disease Control and Prevention
CAAE	Certificate of Presentation for Ethical Consideration
