# Supplementary material for: Determinants of Medication Storage and Disposal Practices in Brazil: A Cross-Sectional Study with Implications for Environmental Exposure
Source: Epidemiologia (Basel). 2026 Jun 29;7(4):90. doi: 10.3390/epidemiologia7040090 (PMC13397877; doi:10.3390/epidemiologia7040090)
Supplement: Supplementary file 1 [file epidemiologia-07-00090-s001.zip › epidemiologia-4301395-supplementary.pdf]

Supplementary File S1
Questionnaire Used in the Cross-Sectional Study

This supplementary material contains the complete questionnaire administered to Brazilian adults to assess medication storage practices, disposal behaviors, handling of injectable materials and pharmaceutical packaging, environmental awareness, and prior exposure to guidance regarding pharmaceutical waste management.

Questionnaire

1. Identification
2. Identification document number
3. Email
4. Gender: ( ) Female ( ) Male ( ) Prefer not to answer
5. Age: options with age ranges
6. Educational level: options with levels of education
7. What region of Brazil do you currently reside in? Options with Brazilian regions and an open option for the city of residence
8. How and where do you store your medications at home? Options for locations within the household
9. Do you periodically check the expiration date of your medications? ( ) Yes ( ) No
10. Do you use continuous medications? ( ) Yes ( ) No. If yes, indicate disease category and number of medications used daily
11. Are any of these medications injectable (require needles)? ( ) Yes ( ) No. If yes, where do you dispose of these sharps?
12. Do you keep leftover medications from completed treatments? ( ) Yes ( ) No
13. Where do you dispose of unused and/or expired medications?
14. Where do you dispose of medication packaging materials?
15. Have you ever received guidance on how to properly store and/or dispose of medications? ( ) Yes ( ) No
16. Do you believe that improper disposal of medications may harm the environment? ( ) Yes ( ) No
17. Do you believe that the general population has sufficient knowledge regarding proper pharmaceutical waste disposal? ( ) Yes ( ) No

Additional Information

The questionnaire was administered electronically using Google Forms and was available to adults residing in Brazil during the study period. Participation was voluntary and anonymous, and respondents could withdraw at any stage before final submission.
